# Treatment Efficacy and Safety of Tenofovir-Based Therapy in Chronic Hepatitis B: A Real Life Cohort Study in Korea

**DOI:** 10.1371/journal.pone.0170362

**Published:** 2017-01-23

**Authors:** Hyo Jun Ahn, Myeong Jun Song, Jeong Won Jang, Si Hyun Bae, Jong Young Choi, Seung Kew Yoon

**Affiliations:** Department of Internal Medicine, Division of Hepatology and Gastroenterology, College of Medicine, The Catholic University of Korea, Seoul, Korea; Indiana University, UNITED STATES

## Abstract

**Background & Aims:**

We evaluated the efficacy and safety of Tenofovir disoproxil fumarate (TDF)-based therapy in naïve and treatment-experienced chronic hepatitis B (CHB) patients for 96 weeks in Korean real life practice.

**Methods:**

A total of 209 CHB patients with a prescription for TDF at the Seoul and Daejeon St. Mary’s hospitals were enrolled from December 2012 to October 2014. We compared the virological responses and evaluated the renal safety of treatment-naive and treatment-experienced patients.

**Results:**

An overall complete virological response (CVR) was achieved in 80.4% and 84.6% of patients at weeks 48 and 96, respectively. In a subgroup analysis, CVR at week 96 was present in 88.4%, 75.0%, 75.5%, and 83.3% of participants in the lamivudine-resistant (LAM-R) group, adefovir-resistant (ADV-R) group, multidrug-resistant (MDR) group, and suboptimal response group, respectively. In a multivariate analysis, ADV-R, MDR, hepatitis B virus DNA, and hepatitis B e antigen were independent predictors for CVR. With regard to renal safety, diabetes mellitus, cirrhosis, and an initial low estimated glomerular filtration rate were independent factors affecting creatinine elevation (≥0.5 mg/dL). Moreover, two patients with DM and cirrhosis experienced TDF-related Fanconi syndrome.

**Conclusions:**

TDF-based therapy demonstrated sustained viral suppression and favorable safety during a 2-year treatment period. The LAM-R and suboptimal response groups showed comparable efficacy to the naïve group, while the ADV-R and MDR groups were significantly associated with a low CVR. Close monitoring of renal safety should be mandatory when treating CHB patients receiving TDF, particularly those with DM and cirrhosis.

## Introduction

Chronic hepatitis B (CHB) remains a global public burden associated with cirrhosis and hepatocellular carcinoma (HCC) [1,2]. The level of circulating hepatitis B virus (HBV) DNA is strongly correlated with progression to cirrhosis or HCC in CHB infection [3,4]. Therefore, eradicating HBV or suppressing viral replication through effective oral nucleos(t)ide analogues (NAs) is a key strategy against the progression of CHB infection. However, drug resistance is a major issue associated with the long-term use of NAs, particularly those with low potency or a low genetic barrier [5].

Currently, tenofovir disoproxil fumarate (TDF), a nucleotide analogue and potent inhibitor of HBV polymerase, is recommended as one of the first-line agents against CHB worldwide [6,7]. In a randomized, double-blind, controlled clinical trial, TDF showed sustained viral suppression, had favorable safety, and displayed no evidence of resistance development for up to 7 years of treatment [8]. In addition, TDF has shown comparable efficacy in patients with chronic HBV infection who are resistant to lamivudine (LAM), compared to treatment-naïve patients [9,10]. TDF treatment may be effective in patients with prior suboptimal responses to LAM or adefovir (ADV) [10,11]. However, there is relatively little or conflicting information in the literature regarding patients with ADV resistance, prior suboptimal response to entecavir (ETV), and multi-drug resistance (MDR).

The HBV genotype and the route of transmission vary greatly worldwide [12]. In European real life studies, the efficacy and safety of TDF have been demonstrated over a treatment period of 3 years [13,14]. However, there is little information on Asian populations. In Korea, TDF was approved for CHB in December 2012. Thus, there are no long-term data for TDF therapy for CHB in Korean practice; only 1-year follow-up results are currently available [15–17]. Thus, we evaluated the efficacy and safety of TDF-based therapy in naïve and treatment-experienced CHB patients for 96 weeks.

## Materials and Methods

### Study design and patients

**Fig 1:**
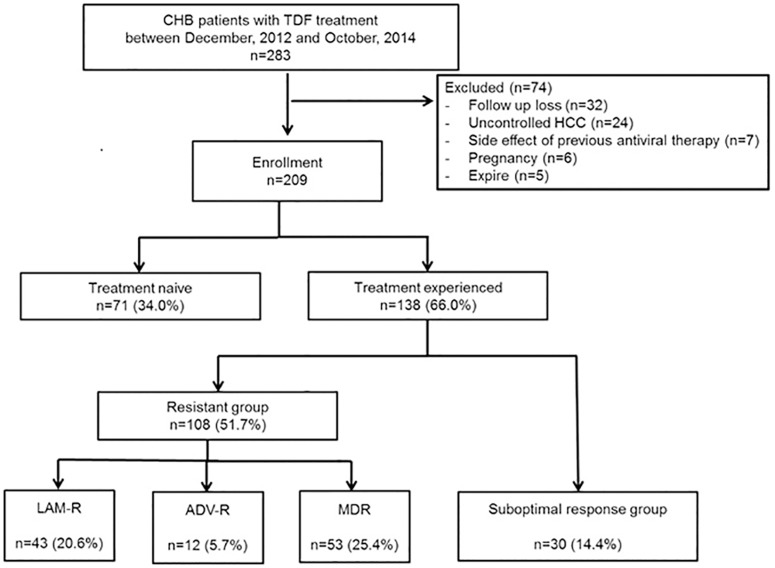
Flow chart of the enrolled participants. CHB, chronic hepatitis B; TDF, tenofovir disoproxil fumarate; HCC, hepatocellular carcinoma; LAM-R, lamivudine-resistant; ADV-R, adefovir-resistant; MDR, multidrug-resistant.

We conducted a multicenter cohort study at the Catholic Medical Centers (the Seoul and Daejeon St. Mary’s Hospitals). All CHB patients who had a prescription for TDF between December 2012 and October 2014 were enrolled. Patients were included if their serum tested positive for hepatitis B surface antigen (HBsAg) for more than 6 months and were followed up for at least 24 months. Exclusion criteria were as follows: follow-up loss or death, patients co-infected with human immunodeficiency virus (HIV) or hepatitis C virus (HCV), uncontrolled HCC, side effects of previous antiviral therapy, and pregnancy. Among 209 patients included, 71 patients were NA-naïve patients. The remaining 138 patients were divided into four groups, based on treatment experience status as follows: the LAM-resistant (LAM-R) group (n = 43), ADV-resistant (ADV-R) group (n = 12), MDR group (n = 53), and suboptimal response group (n = 30) (Fig 1). In a Cox regression analysis, the suboptimal response group was divided into two groups: those that showed a prior suboptimal response to LAM or ADV or LAM plus ADV (suboptimal group 1, n = 11), and those that showed a prior suboptimal response to ETV (suboptimal group 2, n = 19). MDR was defined as two or more types of resistance for NAs, including ETV resistance. A suboptimal response was defined as failure to achieve a complete virological response (CVR) over at least 48 weeks of treatment after initiation of antiviral therapy.

All patients were followed up every 4–12 weeks throughout the treatment period. Serum HBV DNA, alanine aminotransferase (ALT), creatinine (Cr), and estimated glomerular filtration rate (eGFR) were assessed routinely every 12 weeks during treatment. Hepatitis B e antigen (HBeAg) and hepatitis B e antibody (HBeAb) were tested every 12–24 weeks in patients positive for HBeAg at entry.

Patient records were de-identified and anonymously analyzed. This study was conducted in accordance with the Declaration of Helsinki, and ethics approval was obtained from the ethics review board of the Catholic University of Korea (XC16RIMI0011D).

### Endpoints and definitions

The primary endpoints were CVR and renal safety during the treatment period. The secondary endpoints were a biochemical response, HBeAg seroconversion rates, rate of primary non-response, rate of virological breakthrough, adverse events, and treatment adherence during the treatment period.

CVR was defined as a decrease in serum HBV DNA to levels undetectable by a quantitative polymerase chain reaction (PCR) assay. The lower limit of detection for serum HBV DNA was 116 copies/mL. Primary non-response was defined as a decrease in serum HBV DNA of less than 2 log copies/mL after 6 months of therapy. Virological breakthrough was defined as an increase in HBV DNA of more than 1 log copies/mL compared to nadir.

To assess renal safety, we evaluated the serum levels of Cr (≥0.5 mg/dL) and changes in eGFR during the treatment period. Chronic kidney disease (CKD) was defined as an eGFR < 60 mL/min/1.73 m^2^ or proteinuria ≥ 1+ [18].

### Statistical analysis

Continuous variables with normal distributions are presented as means ± standard deviations and categorical data are presented as counts and percentages. Between-group comparisons of continuous or categorical variables were conducted using the t-test, chi-square test, Fisher’s exact test, or one-way analysis of variance (ANOVA). Serum levels of HBV DNA were initially measured in copies/mL and recorded as log transformations. The cumulative probability of CVR during TDF therapy and the cumulative incidence of HBeAg seroconversion were calculated using the Kaplan—Meier methodology. Time-to-event subgroup comparisons were performed using the log-rank test. The Cox proportional-hazards model was used to identify risk factors affecting CVR and HBeAg seroconversion. Logistic regression analysis was performed to identify risk factors affecting creatinine elevation. Factors with a *P* value < 0.100 in the univariate analysis were entered into the multivariate model, and nonsignificant factors were removed using the backward-selection procedure. Statistical analyses were performed using the SPSS software version 19.0 (SPSS Inc., Chicago, IL, USA). A two-sided P-value < 0.05 was considered to indicate statistical significance.

## Results

### Baseline characteristics of the study population

Baseline characteristics of the 209 patients are shown in Table 1. The median follow up durations of the NA-naïve group and the NA-experienced group were 28 (range, 24–33) months and 30 (range, 24–43) months, respectively (*P* = 0.100). Compared to the NA-experienced group, the NA-naïve group had a significantly lower rate of HBeAg-positive status, significantly higher HBV DNA and ALT levels, and a significantly higher rate of cirrhosis.

**Table 1:** Baseline characteristics of the study population (n = 209).

Characteristics	NA-naïve (n = 71)	NA-experienced (n = 138)	*P-*value
**Age (years), mean ± SD**	50.6 ± 11.9	50.9 ± 10.9	0.883
**Male, n (%)**	48 (67.6)	100 (72.5%)	0.464
**HBeAg positivity, n (%)**	27 (38.0)	108 (78.3)	**< 0.001**
**HBV DNA (log copies/mL), mean ± SD**	6.4 ± 1.6	5.3 ± 1.9	**< 0.001**
**ALT (U/L), mean ± SD**	108.2 ± 192.7	54.9 ± 80.0	**0.028**
**eGFR (mL/min/1.73 m**^**2**^**), mean ± SD**	93.4 ± 28.2	87.6 ± 20.7	0.096
**BMI (kg/m**^**2**^**), mean ± SD**	23.5 ± 2.9	23.8 ± 3.4	0.536
**Cirrhosis, n (%)**	39 (54.9)	30 (21.7)	**< 0.001**
**DM, n (%)**	7 (9.9)	16 (9.5)	0.692
**HTN, n (%)**	13 (18.3)	21 (15.3)	0.581
**CKD, n (%)**	4 (5.6)	12 (8.7)	0.430
**Treatment regimen**			**< 0.001**
** TDF monotherapy, n (%)**	71 (100.0)	69 (50.0)	
** TDF + ETV, n (%)**	0 (0.0)	65 (47.1)	
** TDF + LAM or Ldt, n (%)**	0 (0.0)	4 (2.9)	
**Follow up duration, median months (range)**	28.0 (24–33)	30.0 (24–43)	1.000

NA, nucleos(t)ide analogue; SD-standard deviation; HBeAg, hepatitis B e antigen; HBV, hepatitis B virus; ALT, alanine transaminase; eGFR, estimated glomerular filtration rate; BMI, body mass index; DM, diabetes mellitus; HTN, hypertension; CKD, chronic kidney disease; TDF, tenofovir disoproxil fumarate; ETV, entecavir; LAM, lamivudine; LdT, telbivudine.

The treatment regimen of our study population were as follows. In NA-experienced group, 69 of 138 patients (50.0%) were treated with TDF alone, 65 (47.1%) were treated with TDF and ETV, and 4 (2.9%) were treated with TDF and LAM or telbivudine (Ldt), while in the NA-naïve group, all patients were treated with TDF alone. A detailed description of the treatment regimens according to subgroup is given in S1 Table.

### Previous treatment regimens and resistance profile

Previous treatment regimens for the NA-experienced group are listed in S2 Table. With regard to resistance profile, in LAM-R group, 20 of 43 patients had rtM204I mutation alone, and 23 of 43 patients had rtM204V/I plus rtL180M mutation. In ADV-R goup, 8 of 12 patients had mono resistance mutation, while 4 of 12 patients had dual resistance mutation (S3 Table). Moreover, detailed resistance profile in MDR group is shown in S4 Table.

### Overall treatment responses

**Fig 2:**
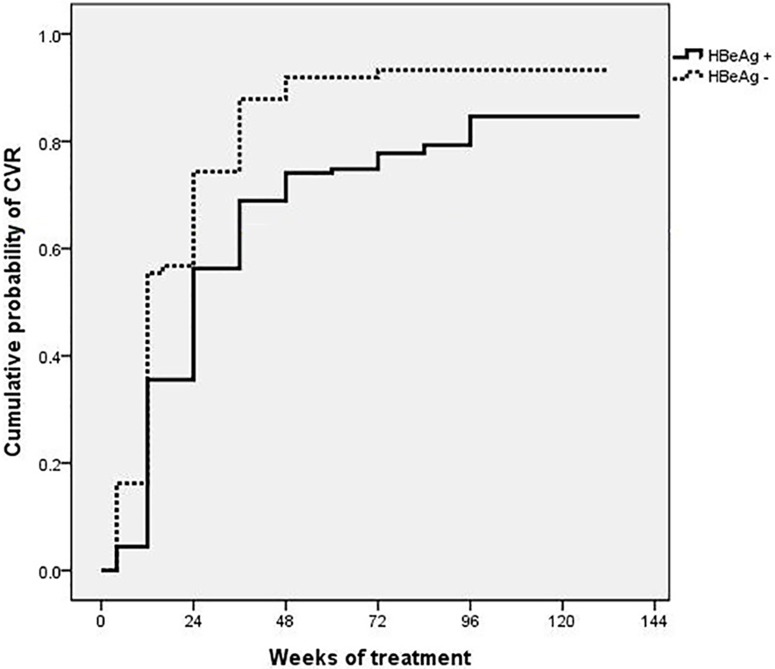
Cumulative rates of a complete virological response (CVR) at week 96 according to HBeAg status. Comparison of CVR between HBeAg (+) patients and HBeAg (-) patients (80.0% vs. 93.2%, *P* < 0.001). HBeAg, hepatitis B e antigen.

The overall CVRs at weeks 48 and 96 were 80.4% and 84.6%, respectively. CVR was achieved in 80.0% of HBeAg (+) patients (n = 135) and in 93.2% of HBeAg (-) patients (n = 74) at week 96 (*P* < 0.001) (Fig 2). HBeAg seroconversion at weeks 48 and 96 was achieved in 5.2% (n = 7) and 11.9% (n = 16) of patients, respectively, while HBsAg loss or seroconversion was achieved in no patient during TDF treatment. Of the 16 patients who achieved HBeAg seroconversion by week 96, 9 were NA-naïve patients (9/27, 33.3%) and 7 were NA-experienced patients (7/108, 6.5%) (*P* < 0.001) (S5 Table).

### Comparison of treatment responses between NA-naïve and NA-experienced group

**Fig 3:**
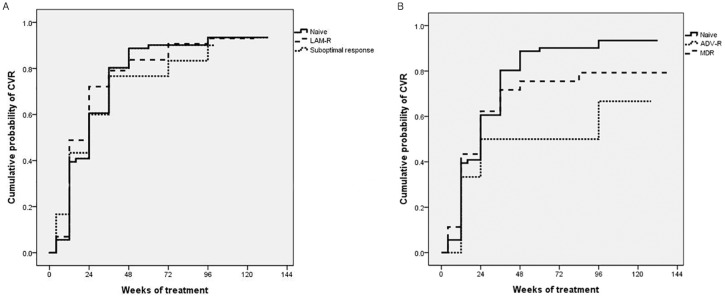
Cumulative rates of a complete virological response (CVR) at week 96 according to subgroup. (A) Comparison of CVR between NA-naïve and LAM-R patients, (91.5% vs. 88.4%, *P* = 0.677) and between NA-naïve patients and the suboptimal response group (91.5% vs. 83.3%, *P* = 0.761). (B) Comparison of CVR between NA-naïve and ADV-R patients, (91.5% vs. 75.0%, *P* = 0.023) and between NA-naïve patients and the MDR group (91.5% vs. 75.5%, *P* = 0.256). NA, nucleos(t)ide analogue; LAM, lamivudine; R, resistant; ADV, adefovir; MDR, multidrug-resistant.

The comparisons of CVR and ALT normalization between the NA-naïve (n = 71) and NA-experienced (n = 138) groups are shown in Table 2. The cumulative probabilities of CVR in the NA-naïve group were 91.9% and 91.5% at weeks 48 and 96, respectively. In the LAM-R and suboptimal response groups, the cumulative probabilities of CVR at weeks 96 were 88.4% and 83.3%, respectively, which did not differ significantly from those of NA-naïve patients, as determined with the log-rank test (Fig 3A). However, in the ADV-R and MDR groups, the cumulative probabilities of CVR at week 96 were relatively low compared to that of the NA-naïve group (75.0%, and 75.5%, respectively) (Fig 3B). Moreover, the ADV-R group (versus treatment-naïve group, hazard ratio [HR]: 0.436, 95% incidence interval [CI]: 0.205–0.925, *P* = 0.031) and the MDR group (versus treatment-naïve group, HR: 0.556, 95% CI: 0.361–0.855, *P* = 0.008) were significantly associated with a low CVR, as assessed in a Cox regression multivariate analysis. Moreover, HBeAg-positive status (HR: 0.730, 95% CI: 0.539–0.988, *P* < 0.001) and initial HBV DNA levels (HR: 0.718, 95% CI: 0.658–0.783, *P* = 0.041) were additional independent predictors of a low CVR during TDF-based treatment (Table 3).

**Table 2:** Comparison of subgroup treatment responses at weeks 48 and 96.

Group	NA-naïve Group (n = 71)	NA-resistant group			Suboptimal response Group (n = 30)
		LAM-R (n = 43)	ADV-R (n = 12)	MDR (n = 53)	
48 weeks					
**CVR (%)**	91.9	85.0	75.0	68.9	68.0
**ALT normalization (%)**	71.8	72.1	41.7	73.6	80.0
96 weeks					
**CVR (%)**	91.5	88.4	75.0	75.5	83.3
**ALT normalization (%)**	88.7	81.4	66.7	73.6	93.3

NA, nucleos(t)ide analogue; LAM, lamivudine; R, resistant; ADV, adefovir; MDR, multidrug-resistant; CVR, complete virological response; ALT, alanine transaminase.

**Table 3:** Univariate and multivariate Cox proportional hazard analyses predicting factors for CVR.

Characteristics	Univariate analysis			Multivariate analysis		
	HR	95% CI	*P-*value	HR	95% CI	*P-*value
**Age, years**	1.000	0.987–1.013	0.985			
**Sex, male**	0.857	0.625–1.174	0.336			
**Cirrhosis**	0.788	0.581–1.070	0.127			
**TDF combination therapy vs. TDF monotherapy**	0.937	0.685–1.282	0.686			
**Treatment status**						
**NA-naïve vs. NA-experienced**	1.128	0.833–1.527	0.437			
**LAM-R vs. NA-naïve**	1.075	0.725–1.593	0.719			
**ADV-R vs. NA-naïve**	**0.517**	**0.247–1.082**	**0.080**	**0.436**	**0.205–0.925**	**0.031**
**MDR vs. NA-naïve**	0.812	0.550–1.198	0.294	**0.556**	**0.361–0.855**	**0.008**
**Suboptimal group 1**^a^ **vs. NA-naïve**	1.014	0.521–1.973	0.967			
**Suboptimal group 2**^b^ **vs. NA-naïve**	0.935	0.548–1.596	0.806			
**Initial ALT, IU/L**	1.000	0.999–1.001	0.990			
**Initial HBV-DNA, log IU/mL**	**0.711**	**0.652–0.775**	**< 0.001**	**0.718**	**0.658–0.783**	**< 0.001**
**HBeAg-positive status**	**0.639**	**0.472–0.864**	**0.004**	**0.730**	**0.539–0.988**	**0.041**

^a^Prior suboptimal response to LAM or ADV or LAM + ADV.

^b^Prior suboptimal response to ETV.

CVR, complete virological response; HR, hazard ratio; CI, confidence interval; TDF, tenofovir disoproxil fumarate; NA, nucleos(t)ide analogue; LAM, lamivudine; R, resistant; ADV, adefovir; MDR, multidrug-resistant; ALT, alanine aminotransferase; HBV, hepatitis B virus; HBeAg, hepatitis B e antigen; ETV, entecavir.

The results of a detailed subgroup analysis of HBeAg seroconversion comparison between the NA-naïve group (n = 27) and the NA-experienced group (n = 108) are shown in S6 Table. The cumulative incidence of HBeAg seroconversion at week 96 in the ADV-R group and the suboptimal response group were 22.2% (*P* = 0.517) and 13.6% (*P* = 0.132), respectively. In the LAM-R and MDR groups, the cumulative incidences of HBeAg seroconversion at week 96 were 0.0% (*P* < 0.001) and 4.5% (*P* = 0.001), respectively. Age (HR: 0.956, 95% CI: 0.914–1.000, *P* = 0.05), the NA-naïve group (versus NA-experienced group, HR: 5.735, 95% CI: 2.134–15.411, *P* = 0.001), and the MDR group (versus NA-naive group, HR: 0.122, 95% CI: 0.026–0.563, *P* = 0.007) were significant factors affecting HBeAg seroconversion at week 96 in a univariate analysis; however, they did not influence HBeAg seroconversion at week 96 in a multivariate analysis (S7 Table).

### Renal safety

To assess renal safety, the NA-experienced group was divided into the ADV-experienced group and the ADV non-experienced group, as ADV is considered a risk factor for renal function impairment [19]. Thus, the three groups were as follows: NA-naïve group (n = 71), ADV-experienced group (n = 64), and ADV non-experienced group (n = 74). The mean eGFRs of the three groups after 96 weeks of treatment were 91.6, 86.9, and 90.6 mL/min/1.73 m^2,^ respectively, similar to the initial eGFR values of 93.4, 84.2, and 90.5 mL/min/1.73 m^2^, respectively. There were no significant differences between the three groups in regard to the mean eGFR during TDF treatment (S8 Table and S1 Fig).

A significant increase in Cr of more than 0.5 mg/dL was reported in seven (3.3%) patients. Of these patients, six had CKD at baseline, six had cirrhosis, three had DM, and five had hypertension. The mean body mass index (BMI) of these patients was 22.2 ± 2.9 kg/m^2^. Furthermore, two had TDF-related Fanconi syndrome. One patient was a 44-year-old female with DM, CKD, and cirrhosis, who developed Fanconi syndrome after 3 months of TDF monotherapy. The other patient was a 70-year-old male with hypertension, DM, and cirrhosis at baseline, who developed Fanconi syndrome 12 months after initiation of TDF and ETV. Both patients were found to have generalized proximal tubular dysfunction in both blood and urine analyses, which is consistent with typical findings of Fanconi syndrome [20]. Fortunately, the renal function of these patients recovered well after cessation of TDF. One patient recovered after conservative management (including solondo 30mg per day for 1month), and she is now treated with baraclude 0.5mg per day. Other patient recovered after intermittent hemodialysis (3 times) and conservative management (including solondo 30mg per day for 1 month), and he is now treated with baraclude 0.5mg q 72 hours due to lowered renal function. In a logistic regression analysis to predict factors associated with a Cr increase of > 0.5 mg/dL, DM (odds ratio [OR]: 48.425, 95% CI: 1.928–1256.556, *P* = 0.018), cirrhosis (OR: 34.365, 95% CI: 1.259–938.149, *P* = 0.036), and initial eGFR levels (OR: 0.901, 95% CI: 0.844–0.961, *P* = 0.002) were independent factors affecting Cr elevation, after adjusting for age, sex, hypertension, CKD, NA-naïve/experienced status, ADV-experienced/non-experienced status, and BMI (Table 4).

**Table 4:** Univariate and multivariate logistic regression analyses predicting factors for creatinine elevation of ≥ 0.5 mg/dL.

Characteristics	Univariate analysis			Multivariate analysis		
	HR	95% CI	*P-*value	HR	95% CI	*P-*value
**Age, years**	1.076	1.001–1.156	**0.046**			
**Sex, male**	0.295	0.064–1.359	0.117			
**Cirrhosis**	**13.238**	**1.561–112.272**	**0.018**	**34.365**	**1.259–938.149**	**0.036**
**TDF monotherapy vs. TDF combination therapy**	0.806	0.152–4.263	0.800			
**DM**	**6.787**	**1.417–32.513**	**0.017**	**48.425**	**1.928–1216.556**	**0.018**
**HTN**	14.828	2.746–80.066	**0.002**			
**CKD**	115.200	12.632–1050.548	**< 0.001**			
**NA-naïve vs. NA-experienced**	1.478	0.322–6.792	**0.001**			
**ADV experienced vs. non-experienced**	0.368	0.043–3.119	0.359			
**BMI**	0.847	0.652–1.099	0.211			
**Initial eGFR**	**0.935**	**0.905–0.967**	**< 0.001**	**0.901**	**0.844–0.961**	**0.002**

HR, hazard ratio; CI, confidence interval; TDF, tenofovir disoproxil fumarate; DM, diabetes mellitus; HTN, hypertension; CKD, chronic kidney disease; NA, nucleos(t)ide analogue; ADV, adefovir; BMI, body mass index; eGFR, estimated glomerular filtration rate.

### Adverse event, primary non-response, virological breakthrough, and treatment adherence

During the study period, new HCC cases occurred in nine (4.3%) patients. The median time to developing HCC was 60 (range, 23–117) weeks after the initiation of TDF-based therapy. Among these patients, eight (88.9%) had cirrhosis. There were two patients who discontinued TDF for TDF-related Fanconi syndrome.

Primary non-response was not observed throughout the TDF-treatment period. Virological breakthrough occurred in six (2.7%) patients, and all cases were associated with poor treatment compliance. In addition, of the eight patients who underwent drug mutation analysis due to virological breakthrough (n = 6) or partial response (n = 2), no patient was confirmed to be resistant to TDF.

## Discussion

In the current study, LAM-R and suboptimal response patients showed comparable efficacy to NA-naïve patients, while ADV-R and MDR were significant factors associated with low CVR. A creatinine increase of ≥0.5 mg/dL above baseline occurred in seven (3.3%) patients, including two cases of Fanconi syndrome, which were associated with DM, cirrhosis, and initial eGFR levels in a multivariate analysis.

CVR in TDF-based therapy at week 96 was achieved in 84.6% of all patients. HBeAg negative status and HBV DNA levels were confirmed as independent predicting factors for CVR in a multivariate analysis, consistent with previous studies [17,21]. Specifically, in NA-naïve patients, CVR was achieved in 91.5% of patients at week 96. According to HBeAg status, CVR was achieved in 85.2% of HBeAg(+) and 95.5% of HBeAg(-) CHB patients at week 96. The rate of CVR in NA-naïve patients in our cohort is comparable to that of previous studies [22,23].

Compared to naïve patients, NA-experienced patients with LAM-R and suboptimal response group did not show the significantly lower probability of CVR. In addition, the majority of patients in both the LAM-R and suboptimal response groups have used TDF monotherapy (67.4% and 86.7%, respectively). In previous studies, the efficacy of TDF monotherapy was proven in patients with LAM-R and those with a prior suboptimal response to LAM or ADV [9,10,24–27]. However, in patients with a prior suboptimal response to ETV, conflicting results have been reported [21,28,29]. In one recent study [21], a suboptimal response to ETV was highlighted as an independent predictor of not achieving a CVR. To clarify the disagreements regarding a suboptimal response to ETV, we subdivided the suboptimal response group into two groups in a Cox regression analysis, namely, a group with a suboptimal response to LAM or ADV and a group with a suboptimal response to ETV. We found that a suboptimal response to ETV was not an independent predictor of a CVR. In other words, the results of our study show that the efficacy of TDF-based therapy was similar in the entire suboptimal response group compared to the naïve group, regardless of whether the previous therapy was LAM, ADV, or ETV. As the majority of the suboptimal response group have used TDF monotherapy (26/30, 86.7%), TDF monotherapy may be preferred for patients with a suboptimal response.

The ADV-R and MDR groups had relatively lower CVRs at week 96 compared to the naïve group, and both groups were significantly associated with a low CVR. TDF and ETV combination therapy was more frequently used than TDF monotherapy in both the ADV-R and the MDR groups (n = 75.0% vs. 16.7% and n = 77.4% vs. 22.6%, respectively). With regard to ADV-R status, previous studies have reported that ADV-R could negatively affect TDF efficacy; our results are in agreement [10,21]. Especially, in ADV-R group with dual resistance genotype (4 of 12), CVR was achieved only in 25%. Although some previous studies have reported that TDF monotherapy is comparable to TDF and ETV combination therapy in CHB ADV-R patients [10,30], long-term follow-up results are needed. Moreover, TDF and ETV combination therapy is generally preferred to TDF monotherapy in MDR CHB patients, and the efficacy of TDF and ETV has been well demonstrated, with variable rates of CVR (~64–86%) [31–34]. A recent 1-year multicenter, randomized, controlled trial conducted in Korea revealed that the CVR of MDR CHB patients with TDF monotherapy versus TDF and ETV combination therapy was not significantly different (71% vs. 73%, P > 0.99) [29]. In the present (2-year) study, the CVR of patients with TDF and ETV did not statistically improve compared to patients with TDF treatment alone (78.0% vs. 66.7%, *P* = 0.247). However, future comparative studies with longer follow-up are required. For now, it can be inferred that TDF and ETV combination therapy may be considered for MDR CHB patients.

The rate of HBeAg seroconversion in NA-naïve patients was 33.3%, while that of the ADV-R (22.2%) and suboptimal response (13.6%) groups at 96 weeks was lower, albeit not significantly. However, in the LAM-R and MDR groups, the cumulative incidences of HBeAg seroconversion at week 96 were 0.0% and 4.5%, respectively (*P* < 0.001 and *P* = 0.001, respectively). Although a low incidence of HBeAg seroconversion in MDR patients has been suggested in previous reports [34,35], it is unclear why no patient in the LAM-R group in the current study showed HBeAg seroconversion. However, both LAM-R status and MDR status were not independent predictors of HBeAg seroconversion in a multivariate analysis.

With regard to renal safety, there was no significant decrease in eGFR in the overall study population. In a subgroup analysis according to ADV experience status, there were no significant differences in Cr clearance between the NA-naïve, ADV-experienced, and ADV-non-experienced groups. However, a significant increase in Cr of ≥0.5 mg/dL occurred in 3.3% of the patients in our cohort. This rate is higher than data from our previous study, in which only 1.7% of patients showed such an increase after 7 years of TDF treatment [8]. Moreover, two patients developed TDF-associated Fanconi syndrome, although they recovered. As DM and cirrhosis were significant predictors of Cr elevation, close monitoring of glomerular and tubular function should be mandatory in CHB patients receiving TDF, particularly in those with DM and cirrhosis.

Our study had several strengths. First, we compared a greater number of groups with the NA-naïve group than previous studies did. Namely, we included subgroups such as the suboptimal response group and the resistant group. Next, our study highlights the importance of paying close attention to the development of renal problems, an issue that was overlooked in a previous 8-year global study. The 2-year timespan of our study demonstrates that renal problems may be of bigger concern than previous thought. The risk factors were similar to those described in the European Association for the Study of the Liver (EASL) guidelines. Finally, in spite of its retrospective design, our study is valuable in that this is first study to report long-term (over 1 year) results of TDF treatment in a cohort of CHB patients in Korea, in which nearly all patients had genotype C, which is associated with a low response to antiviral treatment and poor clinical outcome [36].

In summary, TDF-based therapy demonstrated sustained viral suppression and favorable safety during a 2-year treatment period in Korea. The LAM-R and suboptimal response groups showed comparable efficacy to the naïve group. However, the ADV-R and MDR groups both showed a relatively lower efficacy, and were associated with a low CVR. Close monitoring of renal function may be needed if patients undergoing treatment have DM or cirrhosis.

## Supporting Information

S1 Table Detailed comparison of the treatment regimens according to subgroup. NA, nucleos(t)ide analogue; LAM, lamivudine; R, resistant; ADV, adefovir; MDR, multidrug-resistant; TDF, tenofovir disoproxil fumarate; ETV, entecavir; LdT, telbivudine.
(DOCX)

S2 Table Previous treatment regimens for the NA-experienced group. NA, nucleos(t)ide analogue; LAM, lamivudine; LdT, telbivudine; ADV, adefovir; ETV, entecavir.^1^NAs used > 6 months. Clevudine was considered an LdT.
(DOCX)

S3 Table Detailed genotypic resistance analysis in ADV-R group. ADV, adefovir; R, resistant; TDF, tenofovir disoproxil fumarate; ETV10, entecavir 10mg.*Treated with TDF + lamivudine 100mg.
(DOCX)

S4 Table Detailed resistance profile in MDR group. MDR, multidrug-resistant; TDF, tenofovir disoproxil fumarate; ETV10, entecavir 10mg; ETV, entecavir; R, resistant; LAM, lamivudine; ADV, adefovir.
(DOCX)

S5 Table HBeAg seroconversion rates between the NA-naïve and NA-experienced groups. HBeAg, hepatitis B e antigen; NA, nucleos(t)ide analogue; HBsAg, hepatitis B surface antigen.
(DOCX)

S6 Table Comparison of subgroup serological responses at weeks 48 and 96. NA, nucleos(t)ide analogue; LAM, lamivudine; R, resistant; ADV, adefovir; MDR, multidrug-resistant; HBeAg, hepatitis B e antigen.
(DOCX)

S7 Table Univariate and multivariate Cox proportional hazard analyses to predict factors for HBeAg seroconversion. HBeAg, hepatitis B e antigen; HR, hazard ratio; CI, confidence interval; TDF, tenofovir disoproxil fumarate; NA, nucleos(t)ide analogue; LAM, lamivudine; R, resistant; ADV, adefovir; MDR, multidrug-resistant; ALT, alanine aminotransferase; HBV, hepatitis B virus.†Prior suboptimal response to LAM or ADV or LAM+ADV. ‡Prior suboptimal response to ETV.
(DOCX)

S8 Table Renal safety comparison between subgroups. eGFR, estimated glomerular filtration rate; NA, nucleos(t)ide analogue; ADV, adefovir.
(DOCX)

S1 Fig Renal safety between NA-naïve, ADV-experienced, and ADV non-experienced patients. NA, nucleos(t)ide analogue; ADV, adefovir; eGFR, estimated glomerular filtration rate.
(TIF)
