# Supplementary material for: Treatment Efficacy and Safety of Tenofovir-Based Therapy in Chronic Hepatitis B: A Real Life Cohort Study in Korea
Source: PLoS One. 2017 Jan 23;12(1):e0170362. doi: 10.1371/journal.pone.0170362 (PMC5256915; doi:10.1371/journal.pone.0170362)
Supplement: S1 Table — NA, nucleos(t)ide analogue; LAM, lamivudine; R, resistant; ADV, adefovir; MDR, multidrug-resistant; TDF, tenofovir disoproxil fumarate; ETV, entecavir; LdT, telbivudine. (DOCX) [file pone.0170362.s001.docx]

**S1 Table. Detailed comparison of the treatment regimens according to subgroup.**

| **Group** | **NA-naïve** **group** (n = 71) | **NA-resistant group** | | | **Suboptimal response group (**n = 30) |
| --- | --- | --- | --- | --- | --- |
|  |  | **LAM-R** (n = 43) | **ADV-R** (n = 12) | **MDR** (n = 53) |  |
| TDF monotherapy, n (%) | 71 (100.0) | 29 (67.4) | 2 (16.7) | 12 (22.6) | 26 (86.7) |
| TDF + ETV, n (%) | 0 (0.0) | 11 (25.6) | 9 (75.0) | 41 (77.4) | 4 (13.3) |
| TDF + LAM or LdT, n (%) | 0 (0.0) | 3 (7.0) | 1 (8.3) | 0 (0.0) | 0 (0.0) |

NA, nucleos(t)ide analogue; LAM, lamivudine; R, resistant; ADV, adefovir; MDR, multidrug-resistant; TDF, tenofovir disoproxil fumarate; ETV, entecavir; LdT, telbivudine.
