# Supplementary material for: Treatment Efficacy and Safety of Tenofovir-Based Therapy in Chronic Hepatitis B: A Real Life Cohort Study in Korea
Source: PLoS One. 2017 Jan 23;12(1):e0170362. doi: 10.1371/journal.pone.0170362 (PMC5256915; doi:10.1371/journal.pone.0170362)
Supplement: S2 Table — NA, nucleos(t)ide analogue; LAM, lamivudine; LdT, telbivudine; ADV, adefovir; ETV, entecavir.1NAs used > 6 months. Clevudine was considered an LdT. (DOCX) [file pone.0170362.s002.docx]

**S2 Table. Previous treatment regimens for the NA-experienced group.**

| **Characteristics** | **Total** (n = 138) | **NA-Resistant group** (n = 108) | **Suboptimal response group** (n = 30) |
| --- | --- | --- | --- |
| **Prior NA^1^** |  |  |  |
| LAM or LdT only, n (%) | 22 (15.9) | 21 (19.4) | 1 (3.3) |
| ADV only, n (%) | 11 (8.0) | 6 (5.6) | 5 (16.7) |
| LAM and ADV, n (%) | 38 (27.5) | 33 (30.6) | 5 (16.7) |
| ETV only, n (%) | 35 (25.4) | 22 (20.4) | 13 (76.7) |
| ETV and LAM, n (%) | 9 (6.5) | 9 (8.3) | 0 (0.0) |
| ETV and LdT, n (%) | 2 (1.4) | 0 (0.0) | 2 (6.7) |
| ETV and ADV, n (%) | 12 (8.7) | 9 (8.3) | 3 (10.0) |
| ETV, LAM, and ADV, n (%) | 8 (5.8) | 7 (6.5) | 1 (3.3) |
| ETV, LAM, ADV, and LdT, n (%) | 1 (0.7) | 1 (0.9) | 0 (0.0) |
| **Ongoing treatment at baseline** |  |  |  |
| LAM or LdT, n (%) | 22 (15.9) | 21 (19.4) | 1 (3.3) |
| ADV, n (%) | 12 (8.7) | 6 (5.6) | 6 (20.0) |
| ETV, n (%) | 53 (38.4) | 38 (35.2) | 15 (50.0) |
| LAM + ADV, n (%) | 37 (26.8) | 33 (30.6) | 4 (13.3) |
| LdT + ADV, n (%) | 1 (0.7) | 1 (0.9) | 0 (0.0) |
| ETV + ADV, n (%) | 13 (9.4) | 9 (8.3) | 4 (13.3) |

NA, nucleos(t)ide analogue; LAM, lamivudine; LdT, telbivudine; ADV, adefovir; ETV, entecavir.

^1^NAs used > 6 months. Clevudine was considered an LdT.
