# Supplementary material for: Treatment Efficacy and Safety of Tenofovir-Based Therapy in Chronic Hepatitis B: A Real Life Cohort Study in Korea
Source: PLoS One. 2017 Jan 23;12(1):e0170362. doi: 10.1371/journal.pone.0170362 (PMC5256915; doi:10.1371/journal.pone.0170362)
Supplement: S3 Table — ADV, adefovir; R, resistant; TDF, tenofovir disoproxil fumarate; ETV10, entecavir 10mg.*Treated with TDF + lamivudine 100mg. (DOCX) [file pone.0170362.s003.docx]

**S3 Table. Detailed genotypic resistance analysis in ADV-R group.**

| **Characteristics** | **Total** (n = 12) | **TDF monotherapy** (n = 2) | **TDF + ETV10 Combination therapy** (n = 10) |
| --- | --- | --- | --- |
| **Mono Resistance to ADV (rtA181T/V or rtN236T)** |  |  |  |
| rtA181T/V, n (%) | 6 (50.0) | 1 (50.0) | 5 (50.0) |
| rtN236T, n (%) | 2 (16.7) | 1 (50.0) | 1 (10.0)* |
| **Dual Resistance to ADV (rtA181T/V and rtN236T)** |  |  |  |
| rtA181T/V + rtN236T, n (%) | 4 (33.3) | 0 (0.0) | 4 (40.0) |

ADV, adefovir; R, resistant; TDF, tenofovir disoproxil fumarate; ETV10, entecavir 10mg.

*Treated with TDF + lamivudine 100mg.
