# Supplementary material for: Treatment Efficacy and Safety of Tenofovir-Based Therapy in Chronic Hepatitis B: A Real Life Cohort Study in Korea
Source: PLoS One. 2017 Jan 23;12(1):e0170362. doi: 10.1371/journal.pone.0170362 (PMC5256915; doi:10.1371/journal.pone.0170362)
Supplement: S4 Table — MDR, multidrug-resistant; TDF, tenofovir disoproxil fumarate; ETV10, entecavir 10mg; ETV, entecavir; R, resistant; LAM, lamivudine; ADV, adefovir. (DOCX) [file pone.0170362.s004.docx]

**S4 Table. Detailed resistance profile in MDR group.**

| **Characteristics** | **Total** (n = 53) | **TDF monotherapy** (n = 32) | **TDF + ETV10 Combination therapy** (n = 21) |
| --- | --- | --- | --- |
| **ETV-R** | 42 (79.2) | 31 (96.9) | 11 (52.4) |
| **LAM-R + ADV-R** | 6 (11.3) | 0 (0.0) | 6 (28.6) |
| **ADV-R + ETV-R** | 4 (7.5) | 0 (0.0) | 4 (40.0) |
| **LAM-R + ADV-R + ETV-R** | 1 (1.9) | 1 (3.1) | 0 (0.0) |

MDR, multidrug-resistant; TDF, tenofovir disoproxil fumarate; ETV10, entecavir 10mg; ETV, entecavir; R, resistant; LAM, lamivudine; ADV, adefovir.
