# Supplementary material for: Treatment Efficacy and Safety of Tenofovir-Based Therapy in Chronic Hepatitis B: A Real Life Cohort Study in Korea
Source: PLoS One. 2017 Jan 23;12(1):e0170362. doi: 10.1371/journal.pone.0170362 (PMC5256915; doi:10.1371/journal.pone.0170362)
Supplement: S5 Table — HBeAg, hepatitis B e antigen; NA, nucleos(t)ide analogue; HBsAg, hepatitis B surface antigen. (DOCX) [file pone.0170362.s005.docx]

**S5 Table. HBeAg seroconversion rates between the NA-naïve and NA-experienced groups.**

|  | **Total** (N = 135) | **NA-naïve** (N = 27) | **NA-experience** (N = 108) | ***P*-value** (Log rank test) |
| --- | --- | --- | --- | --- |
| HBeAg seroconversion |  |  |  |  |
| 48 weeks (%) | 7 (5.2) | 4 (14.8) | 3 (2.8) | 0.011 |
| 96 weeks (%) | 16 (11.9) | 9 (33.3) | 7 (6.5) | < 0.001 |
| HBsAg seroconversion (%) | 0 (0.0) | 0 (0.0) | 0 (0.0) |  |

HBeAg, hepatitis B e antigen; NA, nucleos(t)ide analogue; HBsAg, hepatitis B surface antigen.
