# Supplementary material for: Treatment Efficacy and Safety of Tenofovir-Based Therapy in Chronic Hepatitis B: A Real Life Cohort Study in Korea
Source: PLoS One. 2017 Jan 23;12(1):e0170362. doi: 10.1371/journal.pone.0170362 (PMC5256915; doi:10.1371/journal.pone.0170362)
Supplement: S6 Table — NA, nucleos(t)ide analogue; LAM, lamivudine; R, resistant; ADV, adefovir; MDR, multidrug-resistant; HBeAg, hepatitis B e antigen. (DOCX) [file pone.0170362.s006.docx]

**S6 Table. Comparison of subgroup serological responses at weeks 48 and 96.**

| **Group** | **NA-naïve**  **Group** (N = 27) | **NA-experienced group** (N = 138) | | | | | | | |
| --- | --- | --- | --- | --- | --- | --- | --- | --- | --- |
|  |  | **NA-resistant group** | | | | | | **Suboptimal response group** (N = 22) | ***P*-value** (log rank test) |
|  |  | **LAM-R** (N = 33) | ***P*-value** (log rank test) | **ADV-R** (N = 9) | ***P*-value** (log rank test) | **MDR** (N = 44) | ***P*-value** (log rank test) |  |  |
| **HBeAg seroconversion at week 48, n (%)** | 4 (14.8) | **0 (0.0)** | **0.023** | 1 (11.1) | 0.789 | **1 (2.3)** | **0.046** | 1 (4.5) | 0.240 |
| **HBeAg seroconversion at week 96, n (%)** | 9 (33.3) | **0 (0.0)** | **<0.001** | 2 (22.2) | 0.517 | **2 (4.5)** | **0.001** | 3 (13.6) | 0.132 |

NA, nucleos(t)ide analogue; LAM, lamivudine; R, resistant; ADV, adefovir; MDR, multidrug-resistant; HBeAg, hepatitis B e antigen.
