# Supplementary material for: Treatment Efficacy and Safety of Tenofovir-Based Therapy in Chronic Hepatitis B: A Real Life Cohort Study in Korea
Source: PLoS One. 2017 Jan 23;12(1):e0170362. doi: 10.1371/journal.pone.0170362 (PMC5256915; doi:10.1371/journal.pone.0170362)
Supplement: S7 Table — HBeAg, hepatitis B e antigen; HR, hazard ratio; CI, confidence interval; TDF, tenofovir disoproxil fumarate; NA, nucleos(t)ide analogue; LAM, lamivudine; R, resistant; ADV, adefovir; MDR, multidrug-resistant; ALT, alanine aminotransferase; HBV, hepatitis B virus.†Prior suboptimal response to LAM or ADV or LAM+ADV. ‡Prior suboptimal response to ETV. (DOCX) [file pone.0170362.s007.docx]

**S7 Table. Univariate and multivariate Cox proportional hazard analyses to predict factors for HBeAg seroconversion.**

| **Characteristics** | **Univariate analysis** | | | **Multivariate analysis** | | |
| --- | --- | --- | --- | --- | --- | --- |
|  | **HR** | **95% CI** | ***P-*value** | **HR** | **95% CI** | ***P-*value** |
| Age, years | 0.956 | 0.914–1.000 | **0.05** | 0.961 | 0.920–1.004 | 0.072 |
| Sex, male | 0.829 | 0.288–2.386 | 0.728 |  |  |  |
| Cirrhosis | 0.471 | 0.171–1.297 | 0.145 |  |  |  |
| TDF combination therapy vs. TDF monotherapy | 0.308 | 0.088–1.079 | 0.066 |  |  |  |
| Treatment status |  |  |  |  |  |  |
| NA-naïve vs. NA-experienced | 5.735 | 2.134–15.411 | **0.001** | 2.538 | 0.542–11.880 | 0.237 |
| LAM-R vs. NA-naïve | 0.000 | 0.000–1778.382 | 0.963 |  |  |  |
| ADV-R vs. NA-naïve | 0.624 | 0.135–2.890 | 0.547 |  |  |  |
| MDR vs. NA-naïve | 0.122 | 0.026–0.563 | **0.007** | 0.298 | 0.274–13.902 | 0.228 |
| Suboptimal group 1^†^ vs. NA-naïve | 0.452 | 0.057–3.568 | 0.451 |  |  |  |
| Suboptimal group 2^‡^ vs. NA-naïve | 0.361 | 0.078–1.673 | 0.193 |  |  |  |
| Initial ALT, IU/L | 1.001 | 0.999–1.003 | 0.251 |  |  |  |
| Initial HBV-DNA, log IU/mL | 1.139 | 0.883–1.470 | 0.317 |  |  |  |

HBeAg, hepatitis B e antigen; HR, hazard ratio; CI, confidence interval; TDF, tenofovir disoproxil fumarate; NA, nucleos(t)ide analogue; LAM, lamivudine; R, resistant; ADV, adefovir; MDR, multidrug-resistant; ALT, alanine aminotransferase; HBV, hepatitis B virus.

†Prior suboptimal response to LAM or ADV or LAM+ADV. ‡Prior suboptimal response to ETV.
