# Supplementary material for: Treatment Efficacy and Safety of Tenofovir-Based Therapy in Chronic Hepatitis B: A Real Life Cohort Study in Korea
Source: PLoS One. 2017 Jan 23;12(1):e0170362. doi: 10.1371/journal.pone.0170362 (PMC5256915; doi:10.1371/journal.pone.0170362)
Supplement: S8 Table — eGFR, estimated glomerular filtration rate; NA, nucleos(t)ide analogue; ADV, adefovir. (DOCX) [file pone.0170362.s008.docx]

**S8 Table. Renal safety comparison between subgroups.**

|  | **Mean eGFR (mL/min/1.73 m^2^)** | | | ***P***-**value** |
| --- | --- | --- | --- | --- |
| **Time** | **NA-naïve** | **ADV-experienced** | **ADV non-experienced** |  |
| **Initial** | 93.4 | 84.2 | 90.5 | 0.078 |
| **4 weeks** | 88.2 | 80.0 | 89.6 | 0.213 |
| **12 weeks** | 85.0 | 84.1 | 89.0 | 0.550 |
| **24 weeks** | 84.7 | 82.2 | 84.5 | 0.780 |
| **36 weeks** | 85.6 | 82.3 | 86.7 | 0.459 |
| **48 weeks** | 86.8 | 84.5 | 86.6 | 0.777 |
| **60 weeks** | 89.4 | 84.5 | 87.9 | 0.479 |
| **72 weeks** | 88.3 | 84.1 | 85.6 | 0.600 |
| **84 weeks** | 88.7 | 85.4 | 87.6 | 0.730 |
| **96 weeks** | 91.6 | 86.9 | 90.6 | 0.457 |

eGFR, estimated glomerular filtration rate; NA, nucleos(t)ide analogue; ADV, adefovir.
