# Supplementary figures and images for: Treatment Efficacy and Safety of Tenofovir-Based Therapy in Chronic Hepatitis B: A Real Life Cohort Study in Korea
Source: PLoS One. 2017 Jan 23;12(1):e0170362. doi: 10.1371/journal.pone.0170362 (PMC5256915; doi:10.1371/journal.pone.0170362)

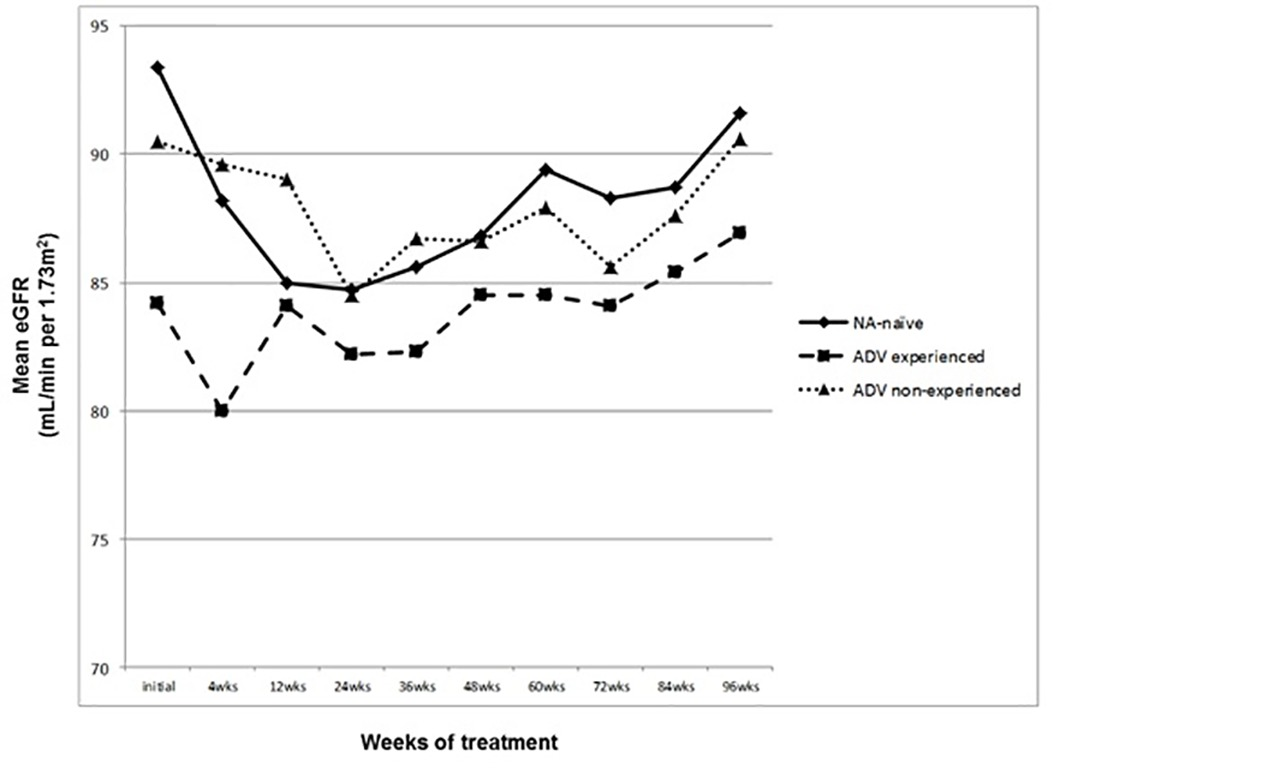

Supplement: S1 Fig — NA, nucleos(t)ide analogue; ADV, adefovir; eGFR, estimated glomerular filtration rate. (TIF) [file pone.0170362.s009.tif]
